# Pilot study of topical imiquimod combined with pembrolizumab for unresectable cutaneous metastatic melanoma

**DOI:** 10.3389/fonc.2026.1797703

**Published:** 2026-06-10

**Authors:** Joseph B. Parker, Richard W. Joseph, Vera J. Suman, Yiyi Yan, Ruqin Chen

**Affiliations:** 1 Department of Internal Medicine, Mayo Clinic Florida, Jacksonville, FL, United States; 2 Department of Hematology and Oncology, Mayo Clinic Florida, Jacksonville, FL, United States; 3 Department of Quantitative Health Sciences, Mayo Clinic, Rochester, MN, United States

**Keywords:** Imiquimod, immunotherapy, metastatic melanoma, pembrolizumab, pilot study

## Abstract

Cutaneous metastases occur in up to 20% of patients with advanced melanoma and can be difficult to manage with systemic therapy alone. Pembrolizumab is a standard therapy with limited local treatment options for cutaneous manifestations. Imiquimod has shown activity in other skin cancers but is less well studied in melanoma. We conducted a pilot trial to evaluate the feasibility, safety, and preliminary efficacy of combining topical imiquimod with intravenous pembrolizumab in patients with unresectable melanoma with cutaneous metastases. This was a single-center, open-label, non-randomized interventional study (NCT03276832). Eligible patients received pembrolizumab IV every 21 days plus topical imiquimod 5% applied Monday–Friday each cycle. The primary endpoint was objective response rate per RECIST v1.1. Secondary endpoints included progression-free survival, and safety. In this study, seven patients were enrolled, five were male and two females; one was Hispanic and six were non-Hispanic White. Median treatment exposure was 8 cycles. ORR was 57.1%, with 3 patients (42.9%) achieving complete response, 1 partial response, and 2 stable disease. The 7-month PFS rate was 57.1%. At study closure, 4 patients remained alive without progression, 2 were alive following progression, and 1 had died of disease. Toxicities were mostly grade 1, with fatigue and anemia most common with minimal grade 2 events. The combination of topical imiquimod and pembrolizumab was feasible, safe, and demonstrated promising antitumor activity in unresectable cutaneous melanoma. A 42.9% CR rate in this small elderly cohort supports further evaluation of local immune modulation with systemic PD-1 blockade for cutaneous melanoma.

## Background

Melanoma is the fifth most common cancer in the United States (US) affecting over 100,000 people annually in the US (1). It is the fifth most common cancer in men and sixth most common in women (1). Melanoma most commonly affects sun exposed areas of the skin; however, it can affect mucosal membranes, ocular sites, and acral sites. Approximately 5% are metastatic at time of diagnosis and 18% of stage I and II melanoma becomes metastatic with common sites of metastasis including the brain, lymph nodes, bone, lung, and liver (2, 3). However, approximately 20% of metastatic melanoma patients suffer from cutaneous metastasis (4).

Standard of care (SOC) first line treatment for metastatic melanoma depends on BRAF mutation status but is primarily centered on immune checkpoint inhibition. Current options include combination nivolumab plus ipilimumab, nivolumab plus relatlimab, or anti-PD-1 monotherapy with nivolumab or pembrolizumab (5). For patients with BRAF V600-mutant disease, BRAF/MEK inhibitor combinations represent an additional option, although immune checkpoint inhibitors are often preferred in the first line setting due to durability of response. Of the ICI therapies, Pembrolizumab is often used in the treatment of metastatic melanoma. It acts by blocking the PD-1 receptor on T lymphocytes to activate the immune system against the tumor cells (6). Pembrolizumab monotherapy has shown a robust and durable response with a response rate of 45% upon initial treatment in treatment naïve patients (7). Additionally, pembrolizumab has shown an acceptable safety profile with less than 10% of patients suffering from grade 3 or 4 adverse events (7). This makes Pembrolizumab one of the first line agents for metastatic, unresectable melanoma.

There have been significant advances in the systemic treatment of metastatic melanoma, but there are limited options for cutaneous manifestations of melanoma. Management of cutaneous metastases from melanoma remains challenging and is not well standardized. Surgical excision may be considered for isolated lesions but is often not feasible in patients with multifocal disease. Radiation therapy can provide local control but is limited by toxicity and field constraints. Intralesional therapies, including talimogene leherparepvec (T-VEC) and intralesional interleukin-2, have demonstrated activity in accessible lesions but require repeated procedures and are not universally available. Systemic therapies such as immune checkpoint inhibitors remain the backbone of treatment; however, cutaneous lesions may demonstrate heterogenous or incomplete responses. A previous study showed poorer response rates in patients treated with monotherapy ICI therapy with cutaneous metastases as comparted to those with visceral metastases (8). As a result, there is a need for additional local treatment strategies that can be combined with systemic therapy to enhance both local and systemic disease control.

Imiquimod is a topical immune response modifier that functions as a toll-like receptor 7 (TLR 7) agonist, leading to activation of the innate immune system and downstream enhancement of adaptive immunity. Activation of TLR7 promotes a Th1-polarized immune response characterized by increased production of interferon-alpha and other proinflammatory cytokines, resulting in recruitment and activation of cytotoxic T lymphocytes within the tumor microenvironment. Preclinical studies have demonstrated that TLR 7/8 agonists can enhance the efficacy of immune checkpoint blockade by increasing antigen presentation and T-cell priming. These findings provide a strong biological rationale for combining topical imiquimod with systemic PD-1 inhibition to augment antitumor immune responses, particularly in accessible cutaneous lesions that may serve as sites of immune priming.

Imiquimod is commonly used for basal cell carcinoma, genital warts, and actinic keratosis, however, reports have demonstrated activity in treating cutaneous melanoma. One study in 2000 showed imiquimod 5% induced a complete response in one patient with lentigo malignant melanoma (9). While in 2002, topical imiquimod 5% induced a complete remission in a patient with invasive melanoma, however, the patient did later develop lymph node metastases (10). Also in 2002, a study reported 3 patients with cutaneous metastases treated with topical imiquimod with excellent responses in two of the three patients with the third patient achieving a response when combined with intra-lesional interleukin-2 (IL-2) (11). These studies showed that topical imiquimod is effective at controlling patients with cutaneous melanoma but that it does not prevent distant metastasis.

Topical imiquimod does have clinical activity in cutaneous melanoma metastases, however, it often requires additional immune modulation to achieve a complete response or prevent distant metastasis. There have been studies that demonstrate this principle. A phase I/II study that combined imiquimod with intralesional IL-2 in thirteen patients demonstrated an overall response rate of 50% and a complete response rate of 40% (12). In another study the combination of topical imiquimod with BCG demonstrated complete remission in two of three patients (13). A case series also examined eleven patients with in-transit melanoma and it found 100% of these patients responded to a combination of intra-lesional IL-2, imiquimod, and a topical retinoid (14). All these studies provide evidence that imiquimod is effective at treating local cutaneous melanoma metastases. However, there are high rates of distant failure with using topical imiquimod.

Despite significant advances in systemic therapies, there are still limited options available for cutaneous lesions of metastatic melanoma. This leaves very few options for treatment of cutaneous lesions of melanoma, especially in the cutaneous metastasis subgroup of patients. Given that imiquimod has shown promising results in treatment of cutaneous melanoma in combination with an additional topical immune modulator, it is thought that topical imiquimod could be used to treat cutaneous lesions in combination with a systemic immunomodulator. The study described here performs a pilot study examining the safety and efficacy of combining topical imiquimod with systemic pembrolizumab in the treatment of metastatic melanoma with cutaneous metastasis.

## Methods and materials

### Trial design and oversight

This study was a pilot single center open label non-randomized interventional clinical study (NCT03276832). It planned for 10 participants, however, 7 patients were enrolled due to COVID-19 early discontinuation of trial in a single group assessment. The study was conducted from June 15, 2018, to October 15, 2019. For these patients pembrolizumab was administered intravenously (IV) on day 1 of a 21-day cycle. Imiquimod 5% was administered topically Monday through Friday during each cycle of IV pembrolizumab.

### Inclusion and exclusion criteria

Participants were able to take part in this trial if they were greater than 18 years of age with histologically confirmed unresectable stage IIIB-IVM1c melanoma, had at least one cutaneous lesion amendable to topical imiquimod, had measurable disease by RECIST v1.1, had an ECOG PS of 0 – 1, had no prior PD-1/PD-L1 therapy, and had adequate hematologic, hepatic, and renal function. Participants were excluded if they had active autoimmune disease requiring systemic therapy, prior pembrolizumab or PD-1/PD-L1 therapy, known CNS metastases or carcinomatous meningitis, active infection requiring systemic therapy, pregnancy or breastfeeding, serious uncontrolled comorbidities or psychiatric illness, or recent use of investigational agents, live vaccines, or other cancer therapies within protocol-defined washout periods.

### Outcome measures

The primary endpoint, the ORR was measured using the RECIST v1.1 criteria. The first scan after treatment initiation was performed after completion of cycle four (approximately twelve weeks) and then was subsequently performed every twelve weeks until disease progression is confirmed. Disease progression was defined as meeting the RECIST v1.1 criteria for disease progression and was evaluated every twelve weeks with criteria met with a PR or CR on two consecutive assessments greater than or equal to twelve weeks apart. Additionally, for secondary outcomes the PFS and the safety profile were evaluated. PFS was measured from registration to radiographic progression. The toxicity profile was assessed using the CTCAE v4.0 criteria.

## Results

### Study population

From June 15, 2018, to October 15, 2019, a total of seven patients were enrolled in the study. Of these patients five were male (71%) and two were female (29%), one was Hispanic (14%) and six were non-Hispanic Caucasian (86%). Two patients had stage IIIB/IIIC disease (29%) and five had stage IV (71%; including M1a-M1c disease). Additionally, at the time of diagnosis five patients had metastasis to lymph nodes (71.4%), four had metastases to subcutaneous tissues (57%), three had metastasis to the skin or soft tissue (43%), and one patient had metastasis to the lung (14%). Of this patient cohort two patients had prior adjuvant treatments (28.6%), one received ipilimumab and one received talimogene iaherparepvec (T-VEC). Additionally, one patient (14.3%) received treatment for prior metastatic disease including ipilimumab and nivolumab. All patient demographic and baseline data can be seen in **Table 1**.

**Table 1:** Demonstrates the patient demographics and baseline disease data.

Variable	Number of patients (percentage) N=7
Male	5 (71.4%)
Female	2 (28.6)
Ethnicity	
Hispanic	1 (14.3%)
Non-Hispanic	6 (85.7%)
ECOG Performance Status of 0	7 (100%)
Prior adjuvant treatments	
Ipilimumab	1 (14.3%)
Talimogene Inaherparepvec (T-VEC)	1 (14.3%)
Prior metastatic disease treatments	
Ipilimumab + Nivolumab	1 (14.3%)
Sites of metastatic disease	
Lymph Nodes	5 (71.4%)
Subcutaneous Tissue	4 (57.1%)
Soft Tissue/Skin	3 (42.9%)
Lung	1 (14.3%)

Data includes gender, ethnicity, and ECOG performance status. The prior adjuvant and metastatic treatments are included as well as the sites of metastasis.

### Treatment course and outcomes

For this patient cohort the number of treatment cycles ranged from 4 – 14, with a median number of 8 cycles. The overall response rate (ORR) was 57.1%. Three patients (42.9%) achieved complete response (CR), one had partial response (PR), and two maintained stable disease (SD). Additionally, at the close of the study two patients remained alive following disease progression, four patients are alive without disease progression, and one patient expired due to disease progression. Overall, the 7-month PFS rate was found to be 57.1% (95% CI:30.1-100%). The outcomes and treatment courses for each patient can be seen in **Table 2**.

**Table 2:** Sociodemographic data, location, overall response, duration of therapy received, reason for discontinuation, and duration of response for each patient enrolled in the pilot study.

Patient number	Age	Gender	Race	Location of disease	Best overall response	Duration of therapy (Months)	Reason for discontinuation	Duration of response (Months)
Patient 1	87	Male	White/Nonhispanic	Skin/Lymph Node	CR	2.2	Side effects, fatigue	18.2
Patient 2	72	Female	White/Nonhispanic	Skin/Lymph Node	SD	7.5	Disease progression	NA
Patient 3	85	Male	White/Nonhispanic	Skin/Lymph Node	CR	3.5	Patient preference	14.4
Patient 4	80	Male	White/Nonhispanic	Skin	PD	2	Disease progression	NA
Patient 5	63	Female	White/Nonhispanic	Skin/Lymph Node	CR	6	Disease Progression	3
Patient 6	85	Male	White/Nonhispanic	Skin/Lungs	CR	4.8	Complete response	14.9
Patient 7	80	Male	White/Nonhispanic	Skin	CR	10.5	Patient preference	41.4

Overall responses included complete response (CR), stable disease (SD), and progressive disease (PD).

### Toxicities

The toxicities that resulted from treatment were predominately grade 1. Grade 1 toxicities included allergic reaction (n=1; 14.3%), anemia (n=3; 42.9%), arthralgia (n=2; 28.6%), hyperbilirubinemia (n=2; 28.6%), fatigue (n=5; 71.4%), and nausea (n=1; 14.3%). Grade 2 toxicities included allergic reaction (n=1; 14.3%), AST elevation (n=1; 14.3%), and hyperbilirubinemia (n=1; 14.3%). Patient toxicity results are shown in **Table 3**.

**Table 3:** Toxicities experienced by the patients enrolled in this pilot study.

Adverse event	Number of patients (Percentage) N=7	
	Grade 1	Grade 2
Allergic Reaction	1 (14.3%)	1 (14.3%)
Anemia	3 (42.9%)	0
Arthralgia	2 (28.6%)	0
Aspartate Aminotransferase (AST) Elevation	0	1 (14.3%)
Bilirubin Elevation	2 (28.6%)	1 (14.3%)
Fatigue	5 (71.4%)	0
Nausea	1 (14.3%)	0

Most adverse events were grade 1, with no grade 3 or 4 toxicities reported.

## Discussion

Immunotherapy has greatly improved the outcomes and quality of life in those with unresectable metastatic melanoma. However, 20% of these patients will still suffer from surgically unresectable cutaneous disease. The study presented here is the first study to examine the effects of the combination treatment of topical imiquimod and pembrolizumab, and one of very few that has examined the effects of topical imiquimod in combination with systemic immunotherapy. This study demonstrates that the combination of topical imiquimod and IV pembrolizumab for the treatment of unresectable cutaneous melanoma is safe, feasible, and tolerable. Additionally, it provides promising antitumor activity with encouraging responses.

The use of topical imiquimod for the treatment of cutaneous manifestations of metastatic melanoma has only been studied in case series, retrospective studies, and early-phase clinical trials and has not been studied extensively. However, it has rarely been studied in combination with systemic immunotherapy. In a previous preclinical model, it was demonstrated that the addition of TLR7/8 agonists can increase the efficacy of anti-CTLA4 or anti-PD1 therapies in mouse melanoma models (15). This study demonstrated that tumors started to respond once a TLR7/8 agonist was added in combination to anti-CTLA4 or anti-PD1 therapies, posing the question about the utility of TLR7/8 agonists in combination with immunotherapy for the treatment of melanoma. Additionally, a case series with two patients treated with a combination of ipilimumab and topical imiquimod for treatment of cutaneous manifestations of melanoma was performed (16). In this case series biopsies showed progression of the cutaneous lesions with just the ipilimumab but had improvement after the addition of topical imiquimod. This case series suggested that the imiquimod and ipilimumab combination does have clinical activity and was well tolerated and showed great response in these patients. This raised the question of whether imiquimod could be used to improve outcomes in those with cutaneous manifestations of metastatic melanoma.

Interestingly, in both studies there was an improved response once the TLR 7/8 agonist agent was added. Given results from prior studies our study also examined the combination of systemic immunotherapy, pembrolizumab, in combination with a topical TLR 7/8 agonist, imiquimod, for treatment of cutaneous manifestations of melanoma which showed promising results. The overall response rate of pembrolizumab monotherapy in melanoma with cutaneous metastases has been cited in previous studies to be between 39% and 44% (8, 17). The patients in this cohort saw an ORR and PFS of 57.1% with a 42.9% CR in patients when combining IV pembrolizumab with topical imiquimod. This offers evidence that this combination does provide antitumor activity and does have clinical activity in treating cutaneous manifestations of melanoma. Additionally, toxicities were mild with the majority being grade one and the most common being fatigue. There were no grade three or four toxicities. This provides evidence that this combination not only has promising clinical impacts but is also safe and tolerable in patients.

There are minimal options for patients suffering from cutaneous manifestations of melanoma. This study offers evidence that topical imiquimod in combination with immunotherapy is a feasible treatment regimen to treat cutaneous manifestations of metastatic melanoma. However, this study does have limitations. The first limitation is the small sample size with the study including seven patients. This greatly limits the statistical power of the study as well can affect the treatment effect that was seen. Additionally, there is little variability in the population with only one non-Hispanic Caucasian and only two females in the cohort, limiting the generalizability of the study. However, even with these limitations this study brings forward the feasibility and safety profile of using topical imiquimod while also bringing to light the need to study this treatment regimen further.

Overall, this study has proved that topical imiquimod in combination with pembrolizumab is effective, safe, and feasible in treatment of cutaneous manifestations of metastatic melanoma. Future studies should be conducted to further expand upon this. Future studies could include a bigger cohort that includes a more diverse patient population to make the study for generalizable. Additionally, with a larger population more statistically significant data and conclusions could be drawn. However, even given the small population size of this study it has provided preliminary data that future studies would be warranted and that topical imiquimod is safe and effective when used in combination with systemic immunotherapy.

## Data Availability

The raw data supporting the conclusions of this article will be made available by the authors, without undue reservation.
